# Low-Cost Soil Moisture Profile Probe Using Thin-Film Capacitors and a Capacitive Touch Sensor

**DOI:** 10.3390/s16081292

**Published:** 2016-08-15

**Authors:** Yuki Kojima, Ryo Shigeta, Naoya Miyamoto, Yasutomo Shirahama, Kazuhiro Nishioka, Masaru Mizoguchi, Yoshihiro Kawahara

**Affiliations:** 1 Faculty of Engineering, Gifu University, Gifu 501-1193, Japan; 2 Graduate School of Information Science and Technology, The University of Tokyo, Tokyo 113-8656, Japan; shigeta@akg.t.u-tokyo.ac.jp (R.S.); shirahama@akg.t.u-tokyo.ac.jp (Y.S.); kawahara@akg.t.u-tokyo.ac.jp (Y.K.); 3 Graduate School of Agricultural and Life Sciences, The University of Tokyo, Tokyo 113-8657, Japan; miyamoto@isas.a.u-tokyo.ac.jp (N.M.); nishioka@isas.a.u-tokyo.ac.jp (K.N.); amizo@mail.ecc.u-tokyo.ac.jp (M.M.)

**Keywords:** soil moisture, soil moisture profile probe, printing circuit, capacitive touch sensor

## Abstract

Soil moisture is an important property for agriculture, but currently commercialized soil moisture sensors are too expensive for many farmers. The objective of this study is to develop a low-cost soil moisture sensor using capacitors on a film substrate and a capacitive touch integrated circuit. The performance of the sensor was evaluated in two field experiments: a grape field and a mizuna greenhouse field. The developed sensor captured dynamic changes in soil moisture at 10, 20, and 30 cm depth, with a period of 10–14 days required after sensor installation for the contact between capacitors and soil to settle down. The measured soil moisture showed the influence of individual sensor differences, and the influence masked minor differences of less than 0.05 m^3^·m^−3^ in the soil moisture at different locations. However, the developed sensor could detect large differences of more than 0.05 m^3^·m^−3^, as well as the different magnitude of changes, in soil moisture. The price of the developed sensor was reduced to 300 U.S. dollars and can be reduced even more by further improvements suggested in this study and by mass production. Therefore, the developed sensor will be made more affordable to farmers as it requires low financial investment, and it can be utilized for decision-making in irrigation.

## 1. Introduction

Soil moisture is an important property for agriculture, indicative of how much water is available for plants. Therefore, its measurement can be utilized for decision-making in irrigation [1,2,3]. Continuous in situ measurement of the soil moisture started around 1940 with the electrical resistance method using a gypsum block [4]. Electrodes in the block measure the internal electrical resistance, which is correlated to soil moisture under an equilibrium condition. This method was simple and cheap, but had problems concerning accuracy, e.g., the electrical resistance of gypsum is affected by temperature [5], response of the electrical resistance to changes in soil moisture is slow [6], and relationship between resistance and soil is not universal for a variety of soils [7].

A neutron attenuation probe was developed as a better method to measure in situ soil moisture in the 1950s [8,9]. The neutron probe measures a count of radioactive beams emitted from a source, which is correlated to soil moisture. The method was successful in field measurement [10], but a health hazard problem remained.

The measurement of in situ soil moisture advanced with the discovery of time domain reflectometry (TDR) [11]. TDR measures apparent permittivity of the soil from the time that an electromagnetic pulse takes to go through parallel electrodes inserted into soil. Because the apparent permittivity of water (≈80) is much larger than those of other soil constituents (≈1–12), the apparent permittivity of soil is strongly correlated to soil moisture [12]. The advantages of the TDR method are that it can measure soil moisture and electrical conductivity simultaneously [13], it is not susceptible to temperature and salinity [11], and a variety of sampling volumes is possible by changing the design of the electrodes [14,15]. However, TDR equipment is expensive, e.g., 3000 U.S. dollars or more.

The capacitance soil moisture sensor is known to be a good alternative to TDR [16,17,18,19]. Soil capacitance depends on the apparent permittivity of soil such that capacitance sensors can determine soil moisture as well as TDR [20]. It has been reported that the capacitance sensors are relatively sensitive to soil temperature [21], do not work well in some soils [22,23,24], and have a small sampling volume [25]. Even with these disadvantages, a variety of capacitance sensors have been developed and commercialized [1,26,27] because they are less expensive than TDR and sufficiently reliable. For example, the capacitance sensors commercialized by Decagon Devices Inc. (Pullman, WA, USA) are known to be relatively inexpensive and reliable, and these sensors have been widely used in recent scientific research [28,29,30].

Agriculture in Japan is in transition from traditional farming, which relies on the individual farmers’ skills and experiences, to precision farming (also called smart farming). Precision farming utilizes a variety of sensors to monitor and control environmental and crop conditions in order to achieve stable crop production and reduce the excessive use of resources [31]. For precision farming, the soil moisture must be measured at multiple locations and at multiple depths. However, with capacitance sensors, the measurement of soil moisture implies a significant investment. Therefore, precise management of fields and crops with a number of soil moisture sensors, i.e., a soil moisture sensor network, is still impeded by the sensor cost.

There is a trade-off between the cost and accuracy of sensors, i.e., more accurate sensors are more expensive. Most of the currently commercialized soil moisture sensors tend to have good accuracy by sacrificing cost reduction as they have been developed for research use. For agriculture, price must have a higher priority than accuracy, i.e., a low-cost sensor even with a relatively weak accuracy is preferred. The development of low-cost soil moisture sensors has been prevented by the expensive and complicated circuits for the high-frequency measurement of the capacitance as well as by the difficulty of obtaining a flexible sensor design that can accommodate a variety of environment and soil types. However, it is possible for soil moisture sensors to be much less expensive than currently commercialized ones, owing to recent technological developments. Thus, the objective of this study is to develop a low-cost soil moisture sensor with the latest technologies, i.e., coplanar plate capacitors on a film substrate and a capacitive touch integrated circuit (IC). The performance of the developed sensors is also evaluated.

## 2. Materials and Methods

### 2.1. Sensor Development

**Figure 1:**
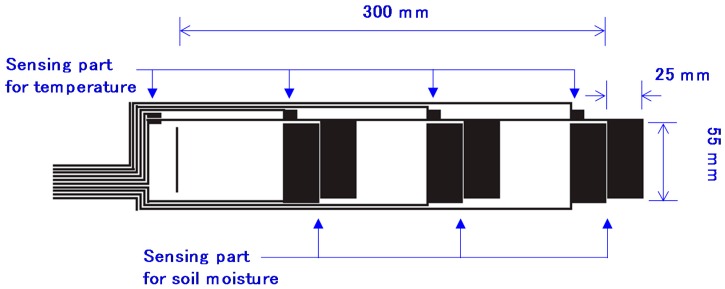
Schematic of copper circuit printed on the film substrate.

**Figure 2:**
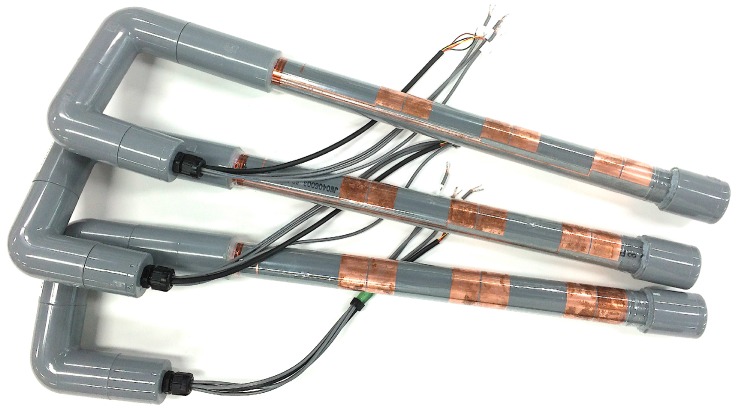
Developed soil moisture sensors.

One of the key technologies for a low-cost soil moisture sensor is film printing. The recent development of printed circuits on film is drawing attention because it has a lower cost than regular electrical circuits and, in particular, it allows for inexpensive, easy, and rapid prototyping such that the cost and time of development can be reduced [32]. A soil moisture sensor can be also made on a film [33,34]. A variety of sensor designs can be examined easily and economically with this technology. Thus, the best design for a soil moisture sensor adaptable to various environments can be developed with low cost. In this study, we developed the copper film substrate shown in Figure 1. The circuit was prepared by etching copper on a polyethylene terephthalate (PET) film. We implemented soil and soil-surface temperature measurement functions on the sensor. Thus, the circuit included sensing parts for soil moisture, sensing parts for temperature, and wiring parts. The circuit had three soil moisture sensing parts to measure the electrical capacitance of soil at 10, 20, and 30 cm depths. These depths were selected to evaluate soil moisture in the root zone for many crops. Soil moisture in deeper soil layers can be measured by developing longer film circuit. Each sensing part consisted of two wide bars, with width and length of 25 and 55 mm, respectively. There was a 1 mm gap between the two bars. These two bars work as a capacitor. Therefore, the sensing area was 25 mm × 55 mm × 2 (two bars) = 2750 mm^2^ (=27.5 cm^2^). The sensing parts for temperature were also located at 10, 20, and 30 cm depth, and there was an extra temperature sensing part 2 cm above the soil surface. The wiring part extended to the end of the film and was connected to a measurement circuit. The film was rolled on a PVC pipe whose outer and inner diameters were 32 and 25 mm, and was covered by a 50 μm (after shrinking) PET heat shrinking film. Before covering it with the PET film, NTC thermistor temperature sensors (NCP18WF104J03RB, Murata manufacturing Company, Ltd., Kyoto, Japan) were pasted on each temperature-sensing part. An edge of the PVC column was filled with a silicone sealant for waterproofing. A photograph of the developed sensors is shown in Figure 2.

The other key technology is a capacitive touch IC for touch sensors. To date, capacitive soil moisture measurement has been made with relatively high frequency, i.e., 70 MHz or higher, in order to obtain good accuracy [27], and the soil moisture sensors are expensive because of the relatively high-frequency capacitance measurement circuit that is required. The recent development of the capacitive touch IC enables us to measure electrical capacitance without investing in complicated circuits. As the capacitive touch IC is being embedded in a variety of devices, such as smartphones and tablets, many low-cost capacitive touch ICs that can be operated with a microcomputer are available. Although the measurement frequency possible with those ICs is lower than with the current commercial soil moisture sensors (e.g., 50 kHz–3 MHz), the cost reduction by using the capacitive touch IC is significant. Thus, using a capacitive touch IC is a good choice for developing a low-cost soil moisture sensor. In this study, a proximity capacitive touch sensor controller MPR121 (NXP Semiconductors N.V., Eindhoven, The Netherlands) was used for measuring the capacitance, and a microcomputer LPC1114 (NXP Semiconductors N.V., Eindhoven, The Netherlands) was used for controlling the MPR121 and for data logging. The MPR121 measures the electric capacitance *C* [F] by the constant current source method [35]. The method runs a constant direct current *I* [A] into the capacitor during a unit time period *t* [s]. There is a relationship between the *C*, *I*, *t*, and voltage *V* [V]:

*V* = *I*·*t*/*C* (1)

With Equation (1), *V* was processed by the analog–digital conversion to determine *C*. A measurement frequency of approximately 62 kHz was used with the developed sensor. This is the default frequency in the MPR121 (MPR121 allows us to choose among 31, 62, and 125 kHz). The reason we decided to use the 62 kHz frequency was because it offers the finest resolution of voltage measurement. The capacitances measured in soils with the developed sensor ranged between 40 and 1000 pF in a preliminary experiment. Given this capacitance range, the voltage measurement resolution was higher at 62 kHz than at the other frequencies. It has been reported that higher frequency is preferred in order to reduce the influence of soil temperature and electrical conductivity [27]; however, those considerations were made for frequencies in the MHz range. The difference between 62 kHz and 125 kHz is small and we concluded that it is more important to obtain a finer resolution than to use a slightly higher frequency.

**Figure 3:**
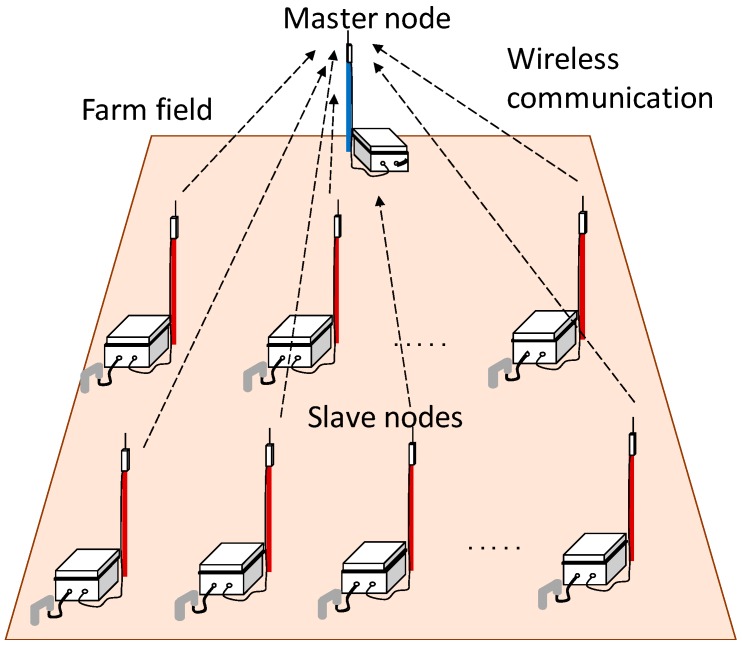
Scheme of the sensor network system with the developed soil moisture sensors.

**Figure 4:**
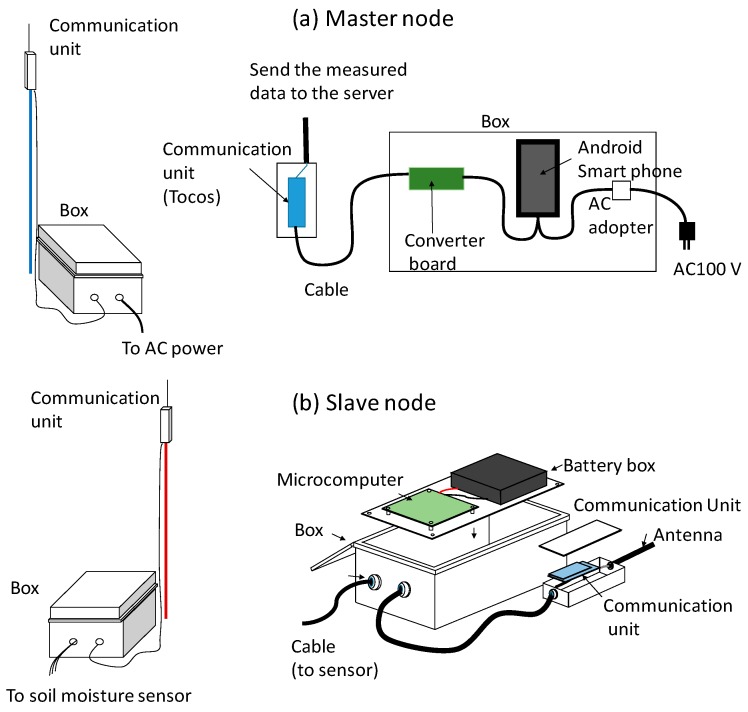
Details of (**a**) master node and (**b**) slave node of the sensor network system.

The developed soil moisture sensors can work as a sensor network system by providing them with a wireless communication function. The sensor network system has multiple sensors with an embedded wireless communication module that are distributed in a field, and their measured data are efficiently collected through wireless communication [36,37]. Figure 3 shows a scheme of this sensor network system. The sensor network system consisted of slave nodes, which were the developed soil moisture sensors that measure the electric capacitance of the soil at multiple points, and a master node, which transfers the measured data into a web server. The details of each node are shown in Figure 4.

The slave node consisted of a microcomputer, a battery box, and a communication unit. The microcomputer and the soil moisture-sensing part were connected by a coaxial cable. The power supply (6 V DC) consisted of four D cell batteries connected in series. The electrical requirement of the slave node is approximately 1 mW, and it is expected that the batteries will last for 1 year. The master node consisted of an Android smartphone, a communication unit, and a converter board. The communication unit in both master and slave nodes used a Tocos strong module (Tokyo Cosmos Electric Co., Ltd., Tokyo, Japan) in the 2.4 GHz band, which allows data transmission over a distance of 1 km. The Android smartphone requires 5 V DC. In this study, we used 100 V AC and an AC adapter for the power supply. The Android smartphone had an inexpensive SIM card embedded, which collects data from the slave nodes and sends the data to the internet server via cellphone communication. A used Nexus 5 (LG Electronics, Seoul, Korea) was purchased and used for the sensor network system in this study. Other low-cost Android smartphones can be an alternative. The measured capacitance data was also stored on the SD card installed in the microcomputer. The master node collected data from the slave nodes and sent them to the server every hour.

### 2.2. Sensor Calibration

The measured electrical capacitance of the soil at each depth needs to be converted to volumetric water content (VWC). VWC is defined as water volume per unit soil volume, and thus, the unit of VWC is m^3^·m^−3^. Usually, empirically obtained equations such as Topp’s equation [11] are used. In this study, we developed our own empirical equations by a calibration experiment. The developed soil moisture sensor was placed in a 50 cm long acrylic column whose inner diameter was 10 cm. The bottom of the column was covered with a fabric membrane. The gap between the sensor and the column was filled with soil sampled at a greenhouse field in Ibaraki prefecture, Japan. The soil was organic-matter-enriched volcanic ash soil (sandy loam), and was packed with a bulk density of 0.8 Mg·m^−3^. Three commercialized soil moisture sensors, 5TE (Decagon Devices, Inc., Pullman, WA, USA), were inserted through the side wall of the column at depths of 10, 20, and 30 cm from the soil surface. The column was placed in a container, which had approximately 5 cm ponded water, and water was infiltrated into the soil from the bottom by capillary force. The ponded water height was manually maintained at 5 cm during the entire experiment.

The electrical capacitance and the VWC at the three depths were measured with both the developed sensor and the 5TEs. The 5TE has been used in various studies as an accurate soil moisture sensor developed for research use. Therefore, the VWC measured with the 5TE was considered as a reference at each depth. Water infiltration continued until the capacitance at the 10 cm depth became constant. This procedure was repeated five times with the same sensor. Preliminary capacitance measurements using the sensor in air and water indicated that the sensor output variability was small, which justified using the same sensor for repeating the procedure. The approximate equations for the three depths were obtained by the least square method from the relationships between the capacitance and VWC. This calibration procedure is easy, fast, and sufficiently reliable.

### 2.3. Sensor Evaluation

**Figure 5:**
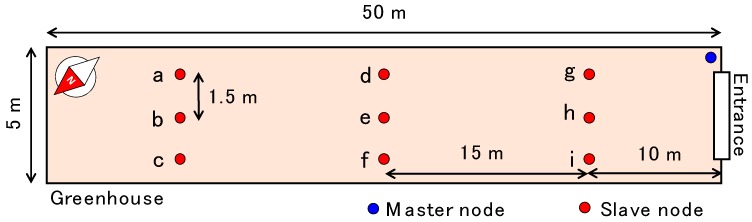
Dimensions of the greenhouse and locations of the master and slave nodes.

The performance of the developed sensor was tested at various fields. In this study, we introduced the data from a grape (*Vitis vinifera* L.) field at the Institute of Sustainable Agro-ecosystem Services, the University of Tokyo (Tanashi, Tokyo, Japan) and from a greenhouse field where mizuna (*Brassica rapa* var. *laciniifolia*) was grown in the Ibaraki Prefecture. The mizuna was planted in the greenhouse during the experiment. The soils at both fields were volcanic ash soil. Four sensors (A, B, C, and D) were installed at the grape field. As the grape field was furrowed, two of them were inserted into a ridge (A, B) and the others were inserted into a furrow (C, D). Nine sensors were installed at the mizuna field to capture the spatial variability of soil moisture in the greenhouse (a–i). The size of the greenhouse and the locations of the soil moisture sensors are shown in Figure 5. Sensor installations were carried out by digging a 35-cm-deep hole with an auger, placing the sensor in the hole, and filling the space with soil from the field. Because the soil was aggregated, it was crushed manually before filling the space. The experimental period was from 15 October to 19 December 2015 for the grape field, and from 12 September to 17 October 2015 for the mizuna field.

## 3. Results and Discussion

### 3.1. Sensor Calibration

**Figure 6:**
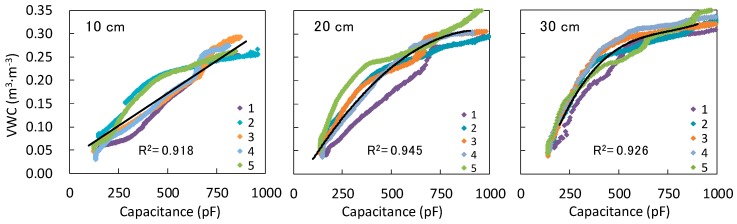
Relationship between volumetric water content (VWC) and electrical capacitance obtained from the calibration experiments. The different colors and numbers indicate the repeated count. The black lines are the approximate equations.

Figure 6 shows the relationship between the VWC measured with the 5TE and the electrical capacitance measured with the developed soil moisture sensors. Five curves with different colors are shown at each depth because the experiment was repeated five times with a same sensor. The VWC increased from 0.03 to 0.35 m^3^·m^−3^, and the capacitance varied between 100 and 1000 pF depending on soil moisture. There was a strong positive correlation between VWC and capacitance. Thus, the developed sensor was able to detect changes in VWC. The five curves did not match well with one another, except at the 30 cm depth. The main reason for the variation in curves at the 10 cm and the 20 cm depths may be that water infiltrated from the bottom reached the 5TE and the developed soil moisture sensor at different times. The 5TE and the sensing part of the developed soil moisture sensor have different sizes and different sampling volumes. The 5TE has a 5-cm-long and 2.5-cm-wide sensing part, which is smaller than that of the developed soil moisture sensor (the developed sensor has a 5.5-cm-long and 5-cm-wide sensing part). It is also assumed that these sensors have different sampling volumes since the sampling volume depends on sensor design and soil conditions [38]. In addition, it was visually observed that the wetting front of the soil was not horizontal during the experiment. Water infiltrated faster where the soil is slightly loose due to a larger hydraulic conductivity than where soil is dense. Thus, the water reached the sampling volume of one of the sensors faster than that of the other. As the elapsed time until water reaches the sensing parts of the sensors at the 30 cm depth was much shorter than at the other depths, the five curves at the 30 cm probably became similar.

As it was difficult to determine which curve is the most appropriate, we derived approximate equations from all sampled data. Based on the curve shape, we concluded that a linear function, a quadratic function, and a cubic function are appropriate for the 10 cm, 20 cm, and 30 cm depths, respectively:

θ = 2.8 × 10^−4^*C* + 3.2 × 10^−2^ at 10 cm depth (2)

θ = −3.7 × 10^−7^*C*^2^ + 7.1 × 10^−4^*C* − 3.5 × 10^−2^ at 20 cm depth (3)

θ = 8.8 × 10^−10^*C*^3^ − 2.0 × 10^−6^*C*^2^ + 1.6 × 10^−3^*C* − 1.4 × 10^−1^ at 30 cm depth (4)

In these equations, θ indicates the volumetric water content and the unit of *C* is pF. The coefficients of determination, R^2^, for 10 cm, 20 cm, and 30 cm depth were 0.918, 0.945, and 0.926, respectively. The sensor accuracies corresponding to the 95% confidence intervals of the model equations were 0.05, 0.03, and 0.02 m^3^·m^−3^, respectively. The reason that each depth had a different curve shape may be the effect of the wiring part on the film substrate. The wiring part in Figure 1 also works as a capacitor that measures a minor capacitance depending on the soil moisture on the wires. The length of the wire is proportional to the depth. Therefore, the relationship between VWC and *C* was linear at the 10 cm depth because there was not a significant capacitance on the short wire. However, the relationship between VWC and *C* became more complex with the larger influence of the capacitance measured on the wiring part at the 20 and 30 cm depths. This might be problematic in field measurements, and in future studies it is necessary to find a method to eliminate the effect of the wiring part on the capacitance measurement. Hereafter, the capacitance measured with the developed sensors was converted to VWC with the equations.

### 3.2. Sensor Performance

**Figure 7:**
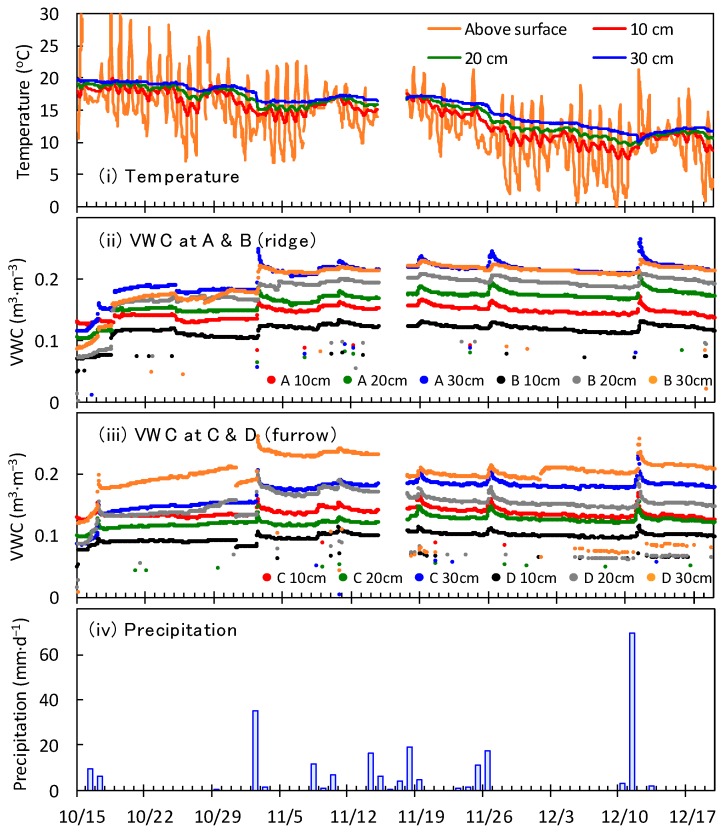
Temperatures above the surface and at 10, 20, and 30 cm depths measured with the developed sensor A (**i**), volumetric water content (VWC) measured in ridges (**ii**), and in furrows (**iii**), and precipitation (**iv**) at the grape field from 25 October to 19 December 2015.

Figure 7 shows the temperature, the VWC measured with the developed sensors at the grape field in Tokyo, and the precipitation rate. The measured VWC initially maintained relatively small values (<0.15 m^3^·m^−3^) after the sensor installation on 14 October, and then increased by 0.01–0.08 m^3^·m^−3^ due to precipitation on 16 and 17 October. The VWC kept increasing after 17 October until 2 November even though there was not a large amount of precipitation. Relative to the value measured on 17 October, the VWC measured just before the precipitation on 2 November had increased by approximately 0–0.03 m^3^·m^−3^. This is due to the improvement in the contact between sensors and soil, i.e., the soil disturbed by the sensor installations settled down slowly, and thus the soil around the capacitors became dense. The contact improvements ceased by the rainfall on 2 November, after which the measured VWC ranged between 0.11 m^3^·m^−3^ and 0.26 m^3^·m^−3^. This indicates that there is some period, two weeks in this case, required for the sensors to start measuring VWC reliably. In addition, the VWC measured with the sensor D suddenly decreased by 0.01–0.03 m^3^·m^−3^ at all depths on 31 October 2015. Weeding was performed at the grape field at this moment, and it was found that a worker had touched the sensor head exposed on the soil surface. It is thought that a space between the sensor and the soil was created, and the contact between them deteriorated by the incident. Clearly, the contact between the sensor surface and the soil is crucial for the developed soil moisture sensors. The measured VWC showed increases due to precipitation and decreases due to evapotranspiration during the daytime after 2 November. The fluctuation of VWC was between 0.11 m^3^·m^−3^ and 0.26 m^3^·m^−3^. There were missed data from 14 November to 17 November due to urgent maintenance of the data logging server. At that time, the system did not have a data resend function in case the data transmission failed (the function was included after this incident). The measured data were stored in a SD card in the slave node, so the missing data can be complemented by collecting data at the field from the SD card. Except for the period with missing data, the VWC measured with the developed sensor was reasonable and similar to those found in other studies [20,30]. The other remark is that the VWC sometimes dropped suddenly to values less than 0.10 m^3^·m^−3^. This phenomenon was most frequently found with sensor D after 3 December. This may be associated with a problem of circuit contact around the capacitance measurement IC. Possibly electrical current leaked somewhere in the circuit and the recorded capacitances were smaller than the real values. The collection rate of regular data during the experimental period was 97.0%.

The measured VWCs tended to be larger with depth, i.e., θ at 10 cm < θ at 20 cm < θ at 30 cm. This is reasonable because the shallow soil layers easily lose water because of their interaction with the atmosphere and are more likely to be loose, so that they cannot hold as much water as the deeper layers. It was expected that the VWCs measured in ridges would be smaller than those measured in furrows because more soil surface is exposed to the atmosphere and the soil is looser in a ridge. However, no clear variation due to differences in location was observed. There might be two reasons for this: (1) the individual differences of sensor output masked the variations in VWC at different locations; and (2) the difference in soil compaction in a ridge location was diminished by the disturbance of soil when the sensors were installed. As the variability of sensor output in air and water was not significant in the preliminary experiment, the individual differences of sensor outputs must be associated with the variability of the contact between each sensor’s capacitors and the surrounding soil. Hence, having good and similar contact conditions at each point is essential to determining the effect of location differences on soil moisture.

**Figure 8:**
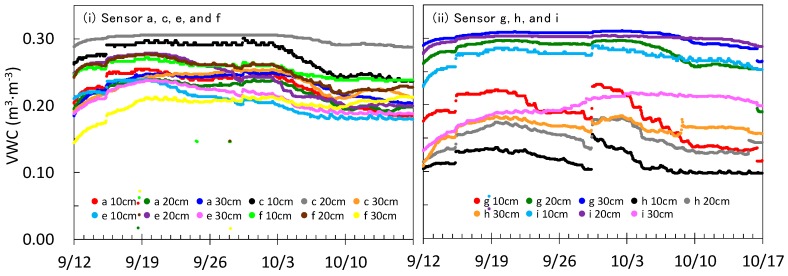
Volumetric water content (VWC) measured with the developed soil moisture sensors at the mizuna green house field from 12 September to 17 October 2015. The left panel (**i**) shows VWC at locations a, c, e, and f, and the right panel (**ii**) shows VWC at locations g, h, and i.

Figure 8 shows VWCs measured with the developed sensors at the mizuna field in Ibaraki. The left panel shows the VWC with sensors a, c, e, and f, located at the middle or north of the greenhouse, while the right panel shows the VWC with sensors g, h, and i, located at the south. The sensors b and d began malfunctioning immediately after irrigation by the sprinklers installed at the ceiling of the greenhouse on 15 September (data not shown). It was found that the communication unit circuits of the sensors b and d were broken owing to water seepage. After sensor installation on 11 September, the measured VWC increased continuously until 20 September. Through this time interval, the measured VWC increased by 0.02–0.07 m^3^·m^−3^ compared to the initial value. It should be noted that there was an irrigation event on 15 September. These observations indicate that the contact between sensor capacitors and soil improved for 10 days, as the sensors at the grape field did. Irrigation was performed twice during the experimental period, on 15 and 29 September. The measured VWC values at most points and depths increased by a maximum of 0.05 m^3^·m^−3^ after the irrigation events. Besides the increases due to irrigation, decreases of VWC due to evapotranspiration were also observed. Therefore, the developed soil moisture sensor satisfactorily detected dynamic changes in VWC.

**Figure 9:**
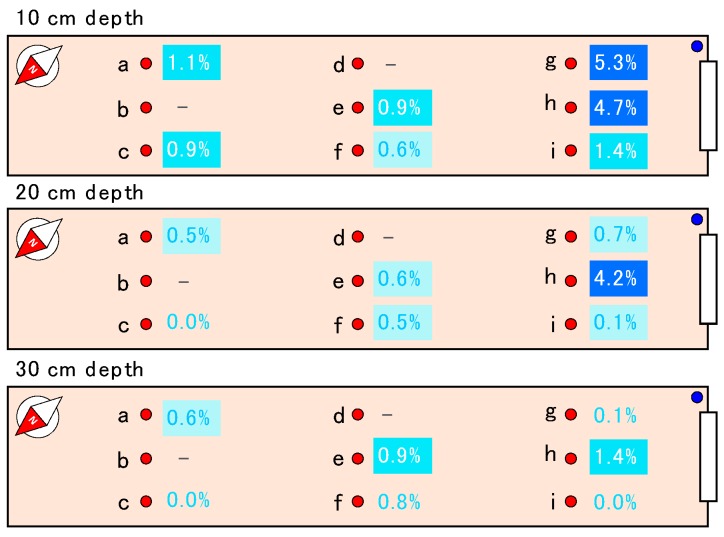
Distribution of the increase in soil moisture at the mizuna greenhouse after irrigation on 29 September 2015.

VWCs in the right panel of Figure 8 (g, h, i) were smaller and changed more dynamically than those in the left panel. For example, the soil moistures at 10 cm at g and h were 0.05–0.15 m^3^·m^−3^ lower than those at other sensor locations, i.e., a, c, e, f, and i. The daily decreases of VWC after the irrigation on 29 September at g and h were sometimes close to 0.02 m^3^·m^−3^, while those at locations a, c, e, f, and i were always less than 0.01 m^3^·m^−3^. Figure 9 shows the increase rates of VWC after the irrigation on 29 September. At 10 cm depth, the VWC at g and h increased dynamically, 5.3% and 4.7%, respectively, but not at the other locations. Only the VWC at h showed a large increase (4.2%) at 20 cm depth, but there was no dynamic change in VWC at the 30 cm depth. As the locations g and h caught plenty of sunshine compared to the other places, the evapotranspiration at these points was enhanced and the soil was relatively dry. This condition probably caused the dynamic increase in VWC at g and h during the irrigation. Therefore, there were relatively large differences (more than 0.05 m^3^·m^−3^) in soil moisture within the mizuna greenhouse field and the developed soil moisture sensor was satisfactory able to detect such variations at the different locations.

Based on the performance evaluations, it seems difficult with the developed sensor to detect precise differences in soil moisture, e.g., less than 0.05 m^3^·m^−3^, at different locations without further improvements to reduce individual differences among sensors. However, the developed sensor can detect relatively large differences (>0.05 m^3^·m^−3^) in a field or among fields, and thus can be utilized to make decisions on irrigation volumes.

### 3.3. Possible Improvements of the Developed Sensor

The developed sensor captured dynamic changes in soil moisture at each depth, but some opportunities for improvement were found though the two experiments. 

The most serious problem of the developed sensor was that the sensor was sensitive to the contact between the sensor capacitors and the soil. The measured soil moisture kept increasing in the beginning of the measurement as contact between the soil and sensors improved due to soil settling, and it took a few weeks for the sensors to show reasonable soil moisture. The differences among individual sensors stem from the variations in contact between the sensor capacitors and the soil. It was observed that the soil moisture measurement suddenly decreased as a result of a human-induced change in the contact between sensors and soils. This problem can be addressed by increasing the frequency of the capacitive touch IC as much as possible. In this study, we used approximately 62 kHz with the MPR121, but this value can be increased up to, for example, 10 MHz by using other capacitive touch ICs that allow us to control the measurement frequency. This will expand the sampling volume of the sensor, which will mitigate the dependence on contact condition between capacitor and soil. The problem may also be solved by changing the sensor design. Because of the current sensor design, the sensor was inserted by digging a hole, placing the sensor in the hole, and filling the gap between the sensor and hole with soil. This installation procedure is the main cause for the sensor needing a few weeks before starting stable soil moisture measurements. A solution would be to develop sensors designed such that they can be hammered into the soil, e.g., sensors with spiky or plate-like shapes. Moreover, as the developed sensors have a sensing part (the film substrate rolled on a PVC pipe), a measurement and data-logging part (the box with a microcomputer and IC), and a communication unit, they require a relatively large space for field installation. This caused workers in the field to accidentally touch the sensors. Therefore, we are developing a new sensor design in which all parts are minimized and combined into a single package. This also will reduce the sensor price and may enable easy waterproofing.

The contact between the film substrate and the PET heat-shrinking film as well as the contact between sensor and soil is considered to be a cause of differences among individual sensors. The PET heat-shrinking film was used to isolate the capacitor from the soil and to hold the substrate on the PVC pipes. Each sensor had a minor difference in the contact between the two films, which was sufficiently small compared to the sensor value. In order to avoid this contact problem, resist ink can be used to isolate the capacitors, instead of the PET heat-shrinking film. It will contribute to making differences between individual sensors negligible.

To address a different issue, a data re-sending function was put in place during this study. With regard to the sudden drops in the measured capacitance, alternatives to the low-cost capacitive touch IC are currently being tested. We have paid less attention to the waterproofing of the communication unit until two of the nine sensors in the Ibaraki greenhouse field malfunctioned due to the irrigation. After that, we have waterproofed the communication unit by taping the slight space between the box and the cap and we have not seen any sensor malfunction due to wetting of the communication unit. Also the communication unit is included inside the waterproofed main box in the new sensor design we are developing, which will render this issue moot.

Another issue we have to investigate in the future is the influence of different types of soils on the relationship between sensor output, i.e., capacitance, and VWC. In this study, the soils in the two fields were similar and, thus, the calibration was only performed once. However, it is known that the relationship between the sensor output and VWC often depends on soil types. Most of the currently commercialized soil moisture sensors are provided with several calibration equations to adapt to a variety of soils, enabling users to choose the appropriate one based on the target soil. The developed sensor must be tested in several different soil types and the impact must be evaluated for expanding the sensor usability. 

## 4. Conclusions

In this study, a low-cost soil moisture sensor using capacitors on a film substrate and a capacitive touch IC was developed, and the performance of the sensor was evaluated in field experiments. Based on the results from the experiments, the developed sensor could capture the dynamic change in soil moisture at 10, 20, and 30 cm depth with some time required after sensor installation for the contact between capacitors to settle down. The measured soil moistures showed the influence of individual sensor differences, and this influence masked minor differences of less than 0.05 m^3^·m^−3^ in soil moisture at different locations. However, the developed sensor could detect large differences of more than 0.05 m^3^·m^−3^ and dynamic changes in soil moisture. Possible improvements to the design of the developed sensor in future were also discussed based on the two field experiments.

The initial cost of a single developed sensor, i.e., the single slave node in Figure 3, was approximately 200 U.S. dollars, and the sale price could be around 300 U.S. dollars. The sale price for the master node is also around 300 U.S. dollars. These costs are much lower than those of soil moisture sensors currently commercialized for research use. With further improvements and mass production, the sensor cost could be reduced even more. This low-cost sensor will therefore be more affordable to farmers as it requires low financial investment, and will thus allow expansion of precision agriculture.
